# Supplementary material for: Alterations in Carbohydrate Quantities in Freeze-Dried, Relative to Fresh or Frozen Maize Leaf Disks
Source: Biomolecules. 2023 Jan 11;13(1):148. doi: 10.3390/biom13010148 (PMC9855396; doi:10.3390/biom13010148)
Supplement: Supplementary file 1 [file biomolecules-13-00148-s001.zip › SupplementalTables_1_6.pdf]

Additional Files

Supplementary Table S1: Chromatography conditions for carbohydrate analysis on the Dionex system. Elution protocols for mono- and di-saccharides and for oligo-saccharides maltose, melibiose, and those of three sugars or greater using a Dionex PA1 column and guard column at a flow rate of 1mL•min^{-1}.

Mono-, and some di-saccharides	Time (min)	Eluant stock solutions: A and D: H_2O; B: 250 mM NaOH; C: 250mM NaOH and 1M NaOAc.	Flow rate mL•min^{-1}
Isocratic	0-40.0	46.4% A; 7.2% B; 0%C; 46.4% D	1.0
Wash	40.1-50.0	25% A; 0% B; 50%C; 25% D	1.0
Wash	50.1-60.0	0% A; 100% B; 0%C; 0% D	1.0
Re-equilibrate	60.1-70.0	46.4% A; 7.2% B; 0%C; 46.4% D	1.0

Melibiose, maltose, and RFOs	Time (min)	Eluant stock solutions: A and D: H_2O; B: 250 mM NaOH; C: 250mM NaOH and 1M NaOAc.	Flow rate mL•min^{-1}
Isocratic	0-30	20.3% A; 58.4% B; 1%C; 20.3% D	1.0
Wash	30.1-35.0	0% A; 0% B; 100%C; 0% D	1.0
Re-equilibrate	35.1-45.0	20.3% A; 58.4% B; 1%C; 20.3% D	1.0

Supplementary Table S2: Carbohydrate and elution time with the conditions related in the Supplementary Table 1. Components of carbohydrate standards for neutral carbohydrates and raffinose family oligosaccharides and elution times on HPLC using a CarboPac PA1 column and guard.

Neutral sugar protocol		RFO sugar protocol	
**Sugar identity**	**Elution Time (min)**	**Sugar identity**	**Elution Time (min)**
Glycerol	1.68	2-deoxyglucose	3.33
myo-Inositol	1.79	Fructose	4.81
Glycerol-O- -D-galactopyranoside	2.33	Glucosamine	6.08
Galactinol	2.54	Sucrose	7.19
Unknown 2	2.72	Melibiose	4.81
Sorbitol	2.89	Raffinose	11.66
Mannitol	3.38	Stachyose	12.55
Trehalose	3.89	Maltose	13.61
2-deoxyglucose	9.68	Verbascose	15.17
Glucosamine	12.74		
Galactose	14.42		
Glucose	15.50		
Fructose	20.10		
Sucrose	25.05		
Melibiose	27.60		

Supplementary Table S3: Chromatography conditions for carbohydrate analysis on the Waters system. Elution protocols for mono- and di-saccharides using Waters hydrophilic interaction liquid chromatography (HILIC) on a Waters Aquity UPLC at 30 °C and a flow rate of 0.35 mL•min^{-1}.

Mono-, and some di-saccharides	Time (min)	Eluant stock solutions: Eluent A: H_2O (Fisher Optima) + 0.1% formic acid (Fisher); Eluent B: Acetonitrile (Fisher Optima) + 0.1% formic acid.	Flow rate mL•min^{-1}

Isocratic	0-1.00	20% A; 80% B	0.35
Linear gradient	1.01<br>to<br>11.00	20% A; 80% B<br>to<br>50% A; 50% B	0.35
Isocratic	11.01-12.50	50% A; 50% B	0.35
Linear gradient	12.51<br>to<br>13.50	50% A; 50% B<br>to<br>20% A; 80% B	0.35
Re-equilibrate	23.50	20% A; 80% B	0.35

Supplementary Table S4: Carbohydrate, retention time, observed ion, and monoisotopic mass with conditions as in Supplementary Table 3. Identities of select carbohydrates analyzed using HILIC separation on the Waters mass spectrometer system, showing retention time, [M-H] ion and the monoisotopic mass of neutral molecule.

**Sugar identity**	**Retention Time (min)**	**Observed ion [M-H]**	**Monoisotopic mass of neutral molecule**
Ribitol	2.66	151.0601	152.0685
Mannitol	3.36	181.0713	182.0790
Glycerol-O- -D-galactopyranoside	3.70	253.0920	254.1002
myo-Inositol	5.14	179.0553	180.0634
Unknown 2	5.88	415.1456	416.1530
Galactinol	6.79	341.1084	342.1162

Supplementary Table S5: Stepwise prediction of glycerol-O- -D-galactopyranoside (GG).

Analysis of Variance

**Source**	**DF**	**Sum of Squares**	**Mean Square**	**F Value**	**Pr > F**
Model	3	1134217	378072	1525.86	<.0001
Error	20	4955.54539	247.77727		
Uncorrected Total	23	1139173			

**Variable**	**Parameter Estimate**	**Standard Error**	**Type II SS**	**F Value**	**Pr > F**
Unknown 2	0.03247	0.00210	59112	238.57	<.0001
Mannitol	-1.05204	0.35832	2135.92000	8.62	0.0082
Fructose	-0.15990	0.02686	8782.69822	35.45	<.0001

Summary of Stepwise Selection

**Step**	**Variable Entered**	**# of Vars In**	**Partial R-Square**	**Model R-Square**	**C(p)**	**F Value**	**Pr > F**
1	Unknown 2	1	0.9820	0.9820	60.4023	1197.94	<.0001
2	Fructose	2	0.0118	0.9938	9.0994	39.84	<.0001
3	Mannitol	3	0.0019	0.9956	2.6360	8.62	0.0082

No intercept in model. All variables left in the model are significant at the 0.05 level. No other variable met the 0.05 significance level for entry into the model.

Model Glycerol-O- -D-galactopyranoside = + 0.03(Unknown 2) - 1.05(Mannitol) - 0.16 (Fructose) + error.

Supplementary Table S6: Stepwise prediction of Unknown 2.

Analysis of Variance					
**Source**	**DF**	**Sum of Square**	**Mean Square**	**F Value**	**Pr > F**
Model	4	3662963775	915740944	5772.92	<.0001
Error	19	3013912	158627		
Uncorrected Total	23	3665977686			
**Variable**	**Parameter Estimate**	**Standard Error**	**Type II SS**	**F Value**	**Pr > F**
Glycerol-O- -D- galactopyra-noside	26.24429	1.74366	35935478	226.54	<.0001
Mannitol	59.98302	10.56455	5113659	32.24	<.0001
Trehalose	-12.86540	4.45628	1322145	8.33	0.0094
Fructose	7.60083	1.11332	7393703	46.61	<.0001

Summary of Stepwise Selection							
**Step**	**Variable Entered**	**# of Vars In**	**Partial R-Square**	**Model R-Square**	**C(p)**	**F Value**	**Pr > F**
1	Glycerol-O- -D- galactopy-ranoside	1	0.9820	0.9820	286.151	1197.94	<.0001
2	Fructose	2	0.0157	0.9977	19.9640	144.54	<.0001
3	Mannitol	3	0.0011	0.9988	3.1452	18.68	0.0003
4	Trehalose	4	0.0004	0.9992	-0.9975	8.33	0.0094

No intercept in model. R^2 is redefined. All variables left in the model are significant at the 0.05 level. No other variable met the 0.05 significance level for entry into the model.

Model Unknown 2 = 26.24(Glycerol-O- -D-galactopyranoside) + 59.98(Mannitol) -12.87(Trehalose) + 7.60(Fructose) + error.
