# Supplementary material for: Alterations in Carbohydrate Quantities in Freeze-Dried, Relative to Fresh or Frozen Maize Leaf Disks
Source: Biomolecules. 2023 Jan 11;13(1):148. doi: 10.3390/biom13010148 (PMC9855396; doi:10.3390/biom13010148)
Supplement: Supplementary file 1 [file biomolecules-13-00148-s001.zip › Supplementary Figures in word.pdf]

Supplementary Figure S1

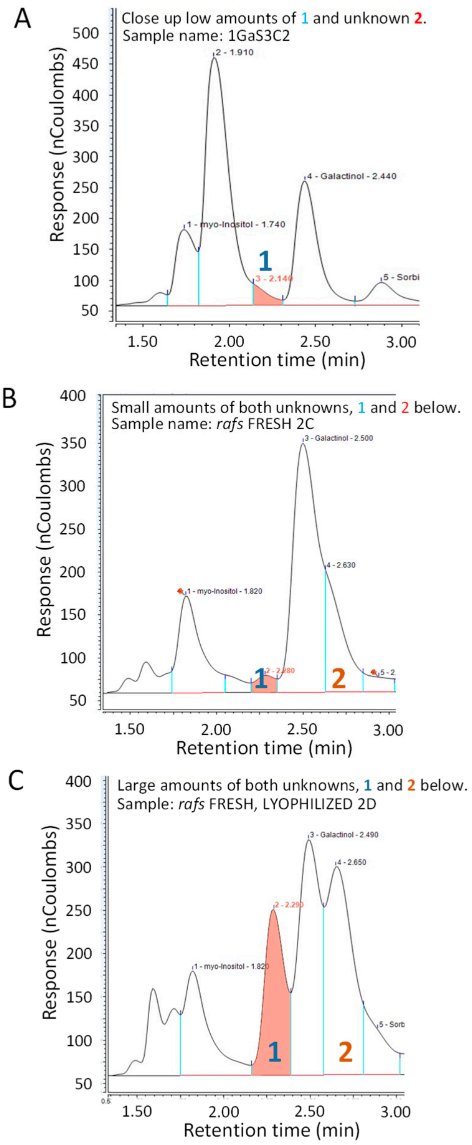

Supplementary Figure S1: Three samples, varying in the amounts of unknowns 1 and 2. Samples were analyzed on the Dionex HPLC system (**A** [little], **B** [more], and **C** [most] of both unknowns; chromatographs shown), and were used to identify these same two peak's elution times on a Waters LC system. The Waters LC was in-line (HILIC) with a Synapt G2 Quadrupole Time-of-flight Mass Spectrograph from which a mass could be captured for both of the unknowns.

Supplementary Figure S2

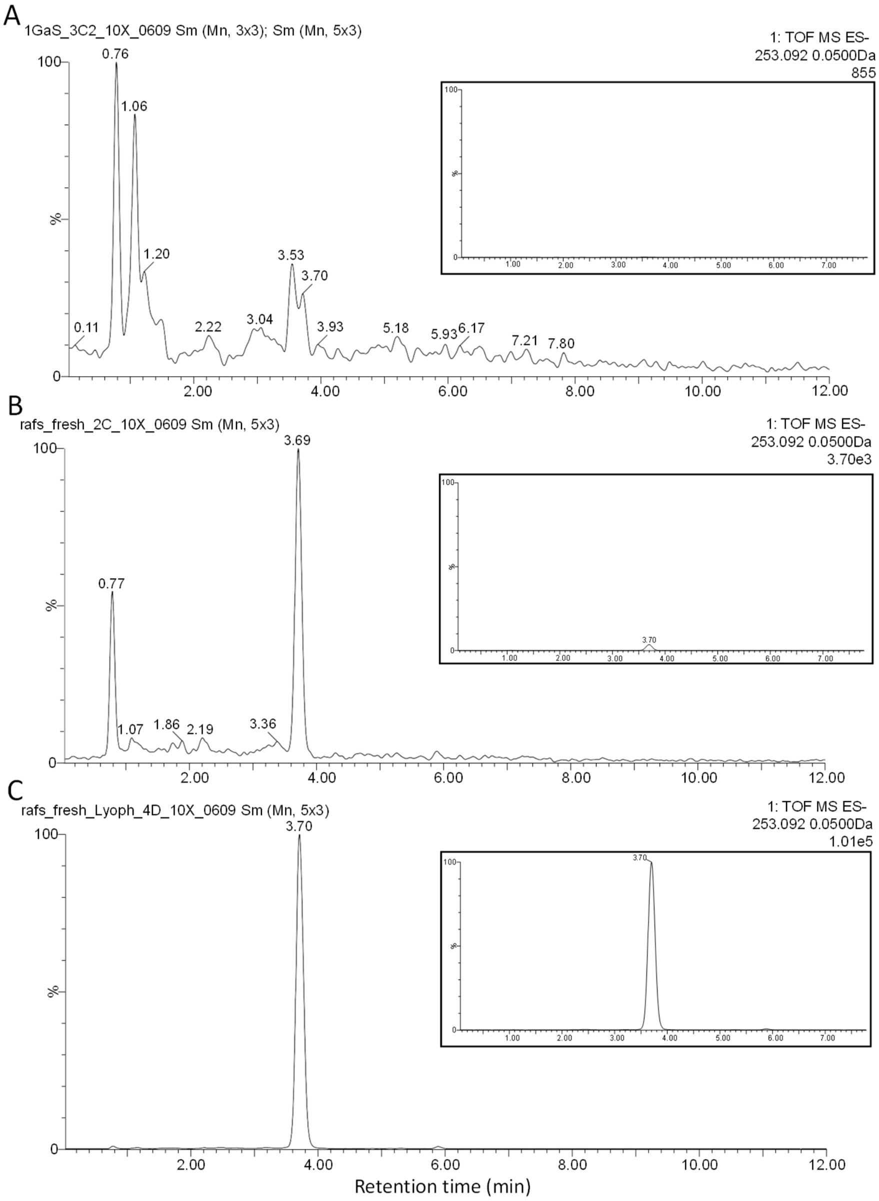

Supplementary Figure S2: Chromatographs of the same samples as in Supplementary Figure S1 using the Waters HILIC system. The Y-axis varies among samples while the insets represent the samples at the same Y-axis scale. The Relative abundances of ion 253.0920, eluting at 3.70 minutes, is reported in negative mode, lacking the mass of a hydrogen.

Supplementary Figure S3

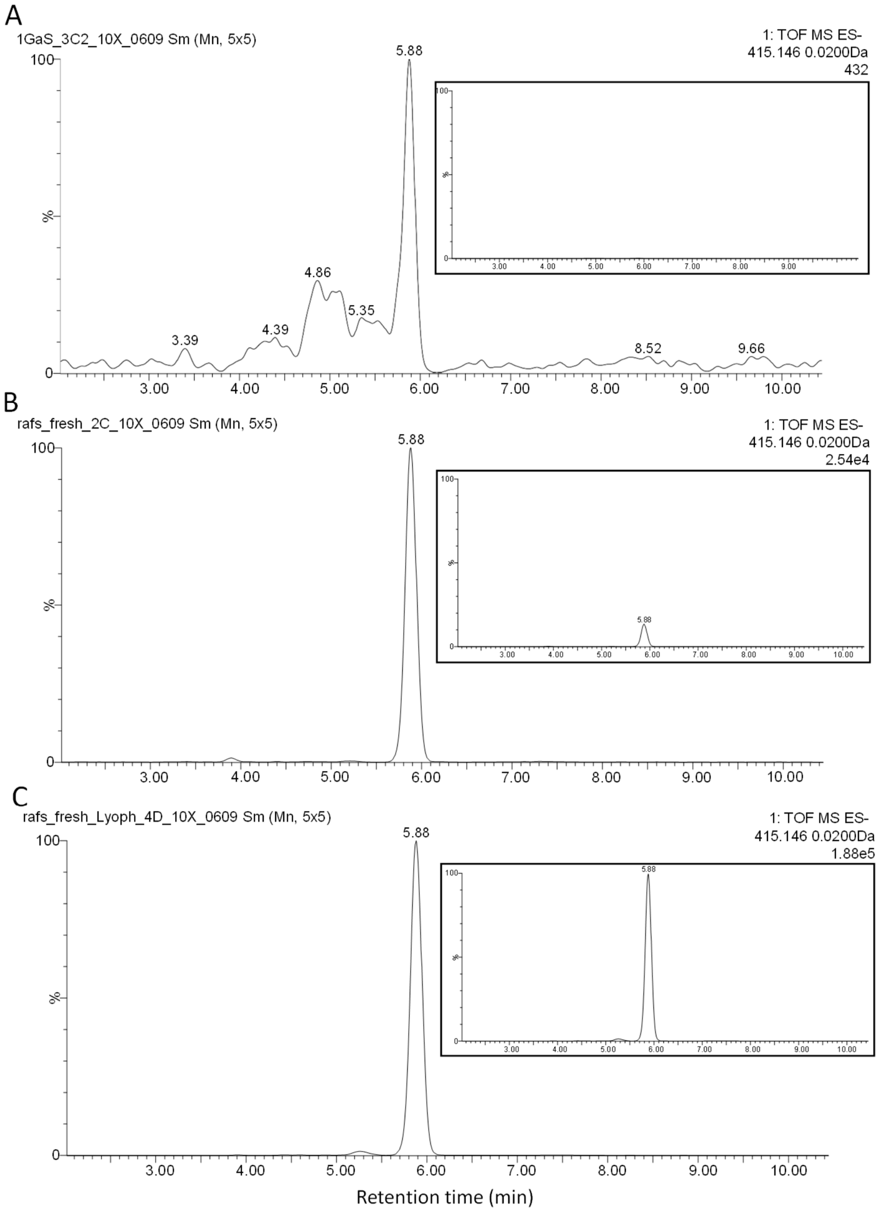

Supplementary Figure S3: Chromatographs of the same samples as in Supplementary Figure S1 using the Waters HILIC system. The Y-axis varies among samples while the insets represent the samples at the same Y-axis scale. The Relative abundances of ion 415.1456, eluting at 5.88 minutes, is reported in negative mode, lacking the mass of a hydrogen.

Supplementary Figure S4

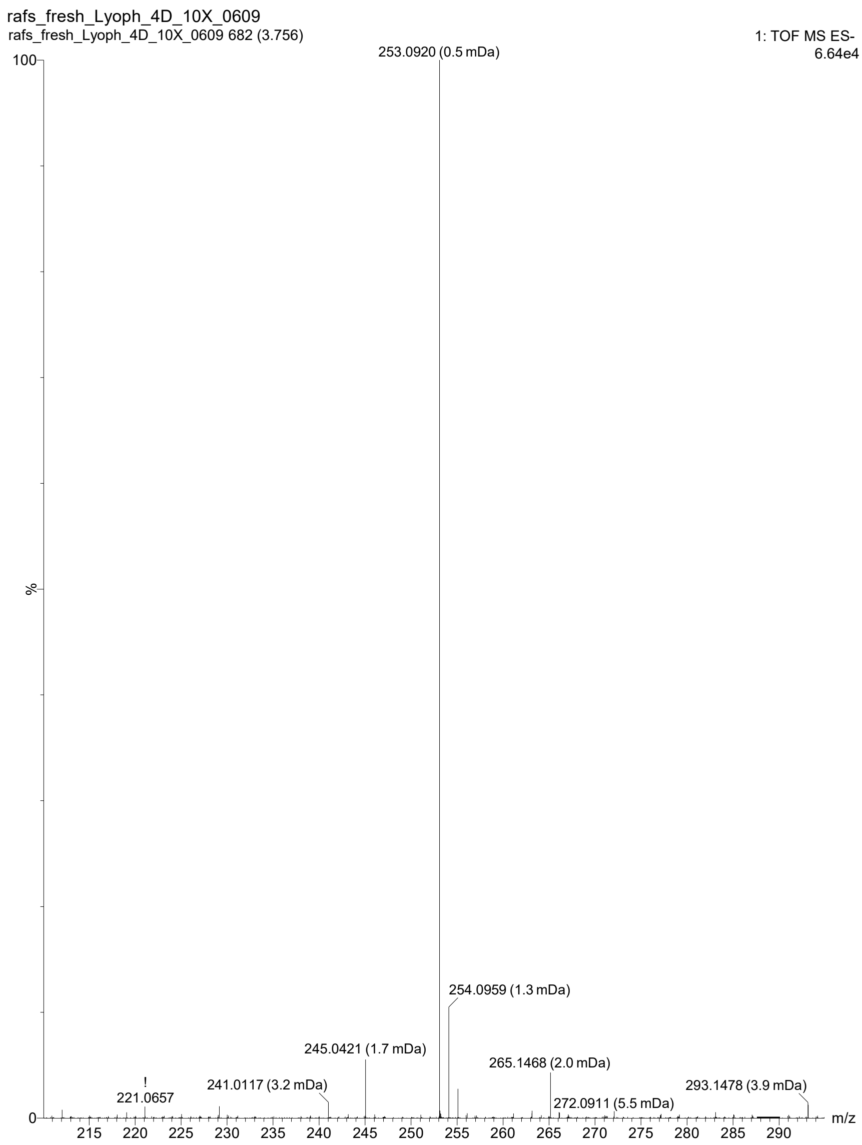

Supplementary Figure S4: Mass of ion at retention time 3.7 min. The lyophilized fresh sample from the *raffinose synthase* mutant was separated using the Waters system for m/z analysis.

Supplementary Figure S5

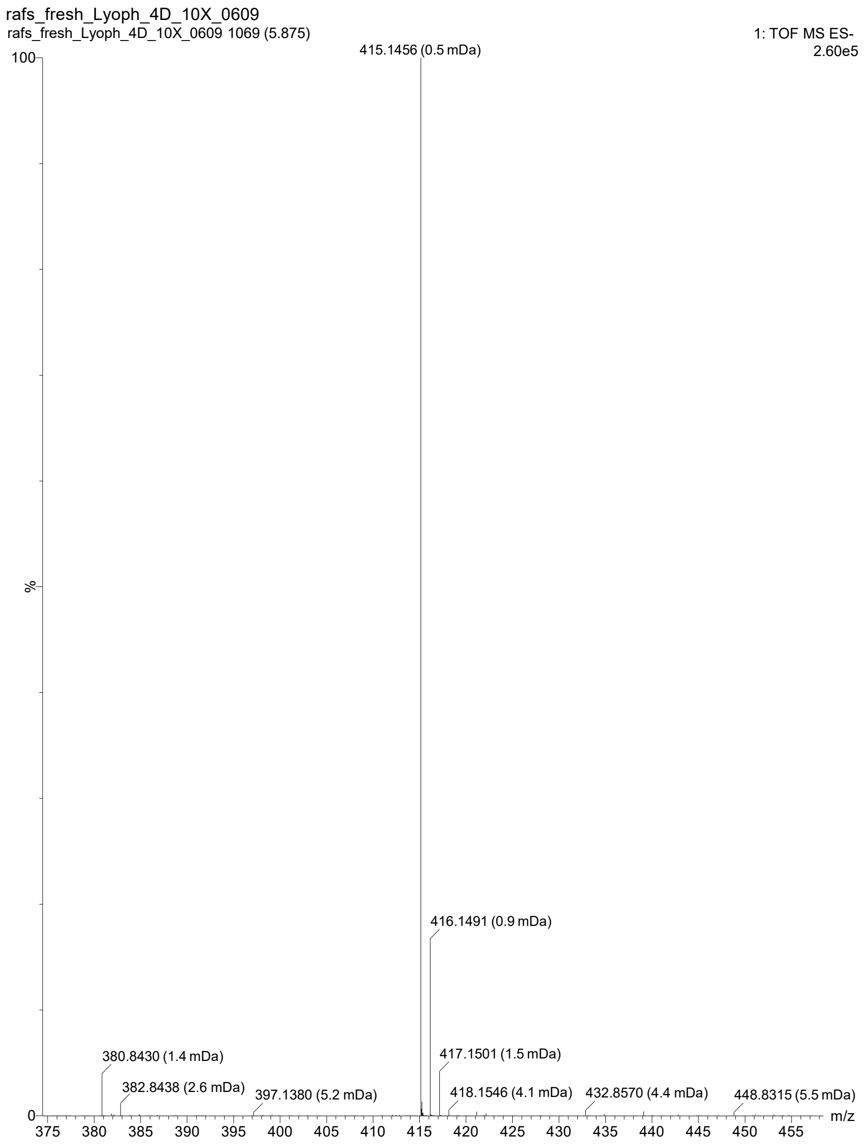

Supplementary Figure S5: Mass of ion at retention time 5.88 min. The lyophilized fresh sample from the *raffinose synthase* mutant was separated using the Waters system for m/z analysis.

Supplementary Figure S6

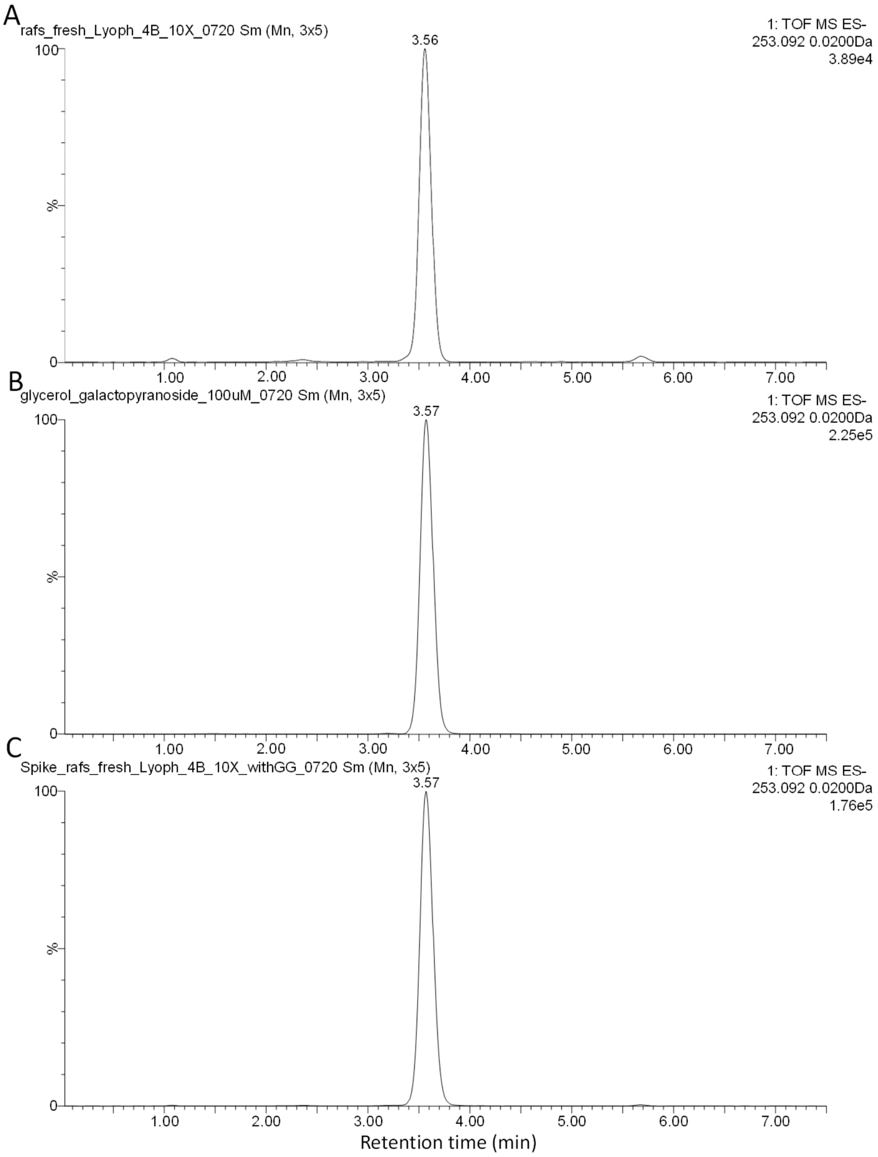

Supplementary Figure S6: Three chromatographs for comparison of elution times of unknown 1 with a commercial source. Chromatographs of the sample with large amounts of unknown 1 spiked (C) or not (A) with a commercial source of the suspected carbohydrate (B) using the Waters HILIC system. The Y-axis varies among samples. The mass of the peak eluting at 3.56 or 3.57 minutes is reported in negative mode, lacking the mass of a hydrogen.

Supplementary Figure S7

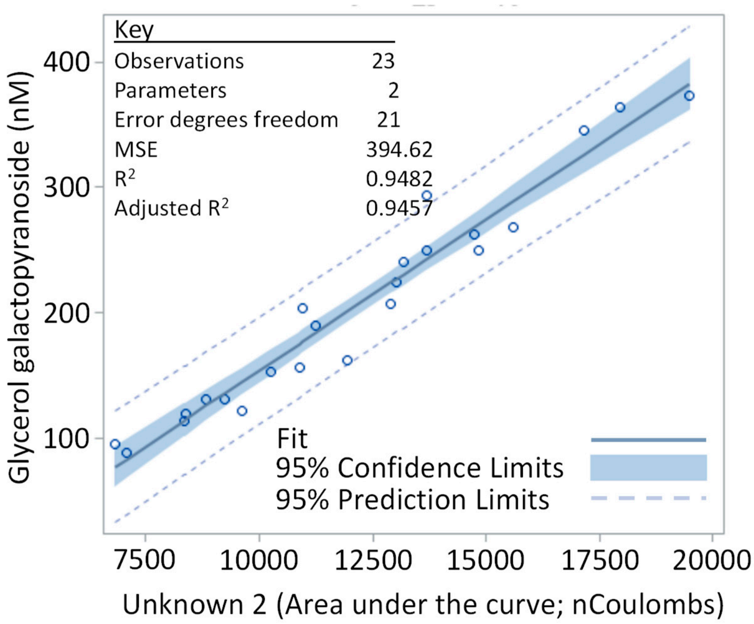

Supplementary Figure S7: Abundance of glycerol-O-b-D-galactopyranoside (GG) and unknown 2 are tightly linked. Linear regression analysis was performed with stepwise addition as described in the Methods.
